# Gustilo IIIC injury of the thigh, MESS score 11, slow oozing of blood on needling the end of the toe, limb preservation successful: A case report

**DOI:** 10.1097/MD.0000000000042162

**Published:** 2025-06-27

**Authors:** Qiushun Zhang, Guangying Zhang, Feng Zhang, Wenzhuo Li, Dongmei Li, Yifeng Zhao

**Affiliations:** a Department of Clinical Medicine, Jining Medical University, Jining, Shandong, P. R. China; b Department of Orthopedics, Dongming People’s Hospital, Dongming County, Shandong, P. R. China; c Department of Orthopedics, Tangta Hospital of Yuncheng, Yuncheng County, Shandong, P. R. China; d Department of Traumatic Orthopedics, Affiliated Hospital of Jining Medical University, Jining, Shandong, P. R. China.

**Keywords:** case report, Ilizarov technique, ischemia, limb salvage, open fracture

## Abstract

**Rationale:**

For limb Gustilo type IIIC injuries with a Mangled Extremity Severity Score (MESS) ≥7, amputation is often necessary. However, due to advancements in surgical techniques, limb preservation is also feasible for some of these patients. There is still no consensus on the reliability of existing scoring systems for determining amputation or limb preservation.

**Patient concerns:**

A patient presented with a Gustilo type IIIC injury of the thigh, absolute indications for amputation (thermal ischemia duration nearly 19 hours, MESS score of 11), but had slow oozing of blood on needling the end of the toe.

**Diagnoses:**

Gustilo type IIIC injury of the thigh with prolonged ischemia and high MESS score.

**Interventions:**

The treatment plan was immediately changed from amputation to limb preservation. The femur was overlapped, the limb was shortened by 5 cm in one stage and fixed with an external frame. The femoral artery defect (up to 6 cm) was managed with direct end-to-end anastomosis after freeing. After viability was confirmed, a second stage lengthened the limb to normal length and internal fixation was performed for the femoral stem fracture.

**Outcomes:**

The limb was successfully preserved, and good lower limb function was achieved.

**Lessons:**

Even for Gustilo type IIIC injuries with a MESS score ≥7, if slow oozing of blood is observed at the end of the needled limb, active limb preservation should be considered. One-stage limb shortening to rapidly restore blood flow and 2-stage lengthening to recover limb length are practical techniques conducive to successful limb preservation.

## 1. Introduction

Historically, the probability of amputation for Gustilo type IIIC injuries with open fractures has been extremely high, and even with very significant advances in trauma management techniques in recent years, the probability of amputation for this type of injury has remained high, at approximately 30% to 50%.^[1–4]^

It is generally believed that a Mangled Extremity Severity Score (MESS) ≤6 indicates a good prognosis and can be treated with limb preservation; an MESS ≥7 points indicates amputation, and most patients need 1-stage amputation.^[5,6]^ However, with the active application of microsurgical vascular repair and composite bone and soft tissue flap grafting techniques, open fractures of the lower extremity with MESS ≥7 points are possibly amenable to limb preservation, especially when plantar sensation is present.^[7]^

In this study, we report a case of successful limb preservation in a patient with Gustilo type IIIC injury of the thigh with a duration of thermal ischemia of nearly 19 hours and an MESS score of 11. The patient eventually had better plantar sensation and achieved good walking, squatting, and stair climbing ability (see Supplemental Videos 1–3, Supplemental Digital Content, https://links.lww.com/MD/O676; https://links.lww.com/MD/O677; https://links.lww.com/MD/O678, which demonstrates the patient’s good recovery of limb function), as reported below.

## 2. Case presentation

### 2.1. Hospitalization

**Figure 1.**
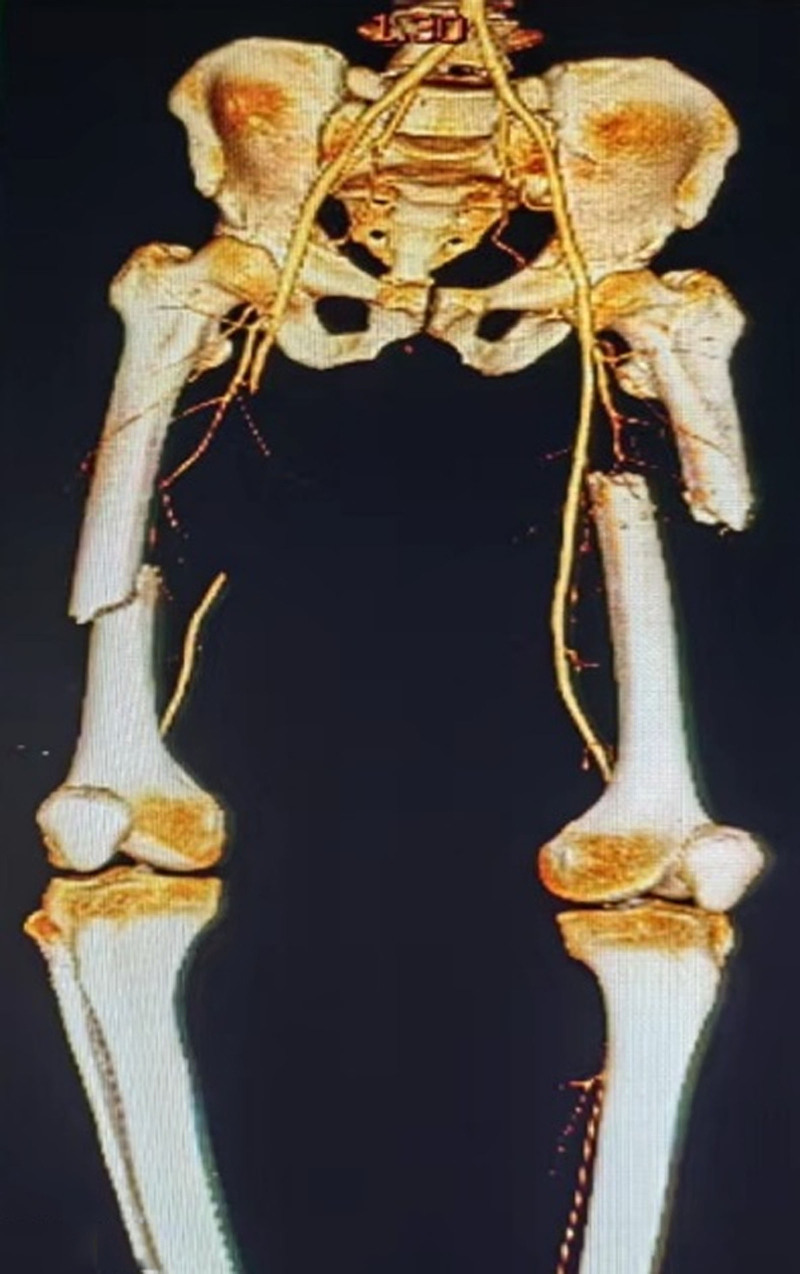
Middle-aged male patient with double femoral shaft fracture due to trauma.

**Figure 2.**
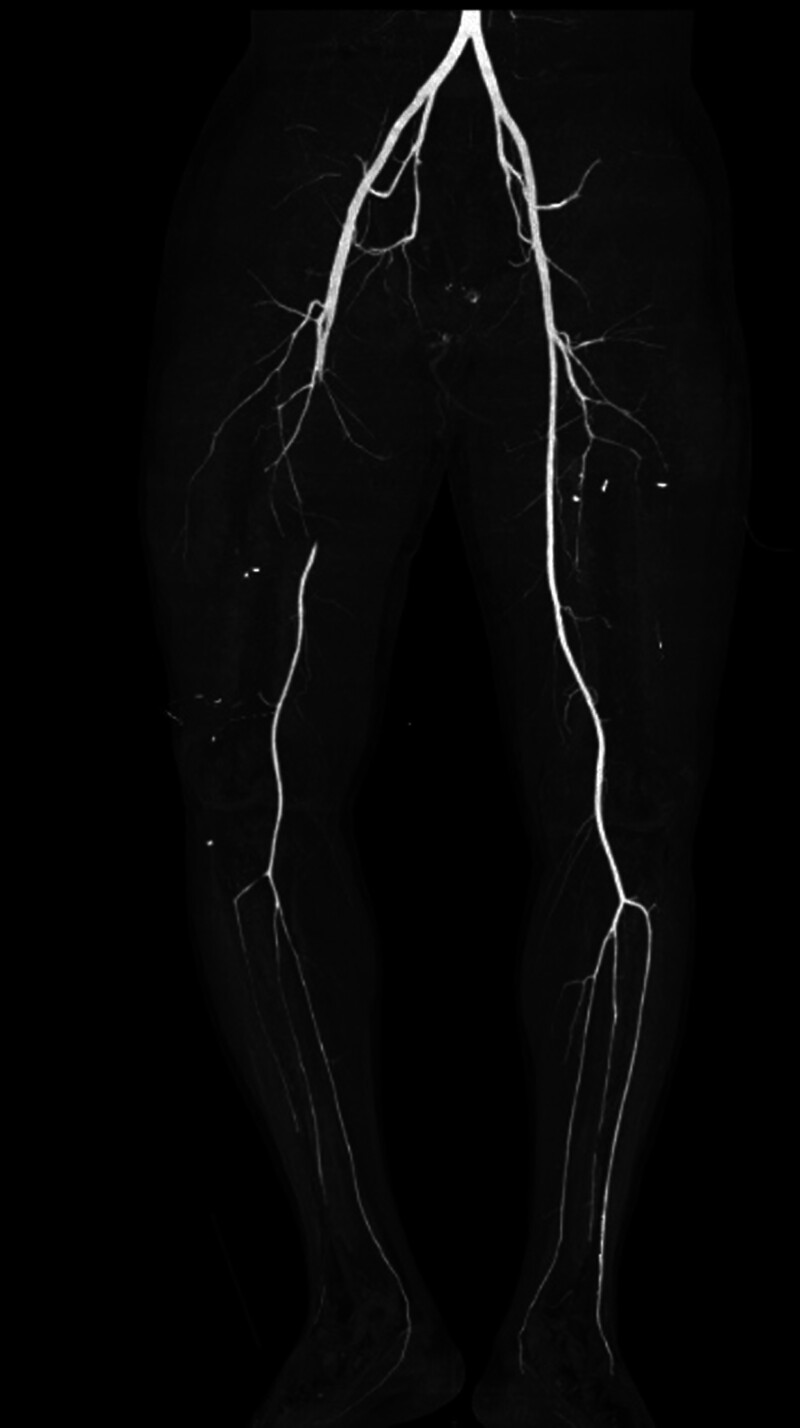
CTA showing a long-segment embolism of the right femoral artery. CTA =

A 46-year-old male, previously fit, was admitted to a local hospital after being crushed by an automobile on both thighs against a wall 16 hours earlier, resulting in an open fracture of the mid-thighs of both thighs. On examination at the hospital, he was unconscious and in a state of shock, and the dorsalis pedis arterial pulsations were accessible and weak. The open wounds on both thighs were debrided and sutured, both lower limbs were externally immobilized with braces, and the patient was admitted to the monitoring room for blood transfusion and other rescue treatments. Shock was corrected, and the patient was then transferred to our hospital at his request 16 hours after the injury. Our hospital examination revealed that the patient was conscious and that his vital signs were stable. Signs of femur fracture of the middle and lower parts of the right thigh were observed. Loss of all deep and superficial sensation in the right lower extremity below the middle and lower part of the limb; complete loss of motor function of the right foot, ankle, and knee; complete loss of dorsal foot artery and posterior tibial artery pulsation of the right foot; low skin temperature of the right foot, pale in color. Signs of fracture in the middle of the left thigh, normal sensation and movement of the left foot, strong pulsation of the dorsal foot artery and posterior tibial artery of the left foot, and good peripheral blood flow. Auxiliary examination (urgent examination after admission to our hospital): computer tomography angiography (CTA) examination of both lower limb arteries, suggesting (1) interruption of the continuity of the lumen of the middle and upper part of the right femoral artery, with a defect of up to 11 cm (Figs. 1 and 2); and (2) fracture of the middle and lower part of the right femur and the middle and upper part of the left femur (Fig. 1). Admission diagnosis: (1) right thigh Gustilo type IIIC injury and (2) left thigh Gustilo type II injury.

### 2.2. Preoperative evaluation

Upon admission to our hospital, after rapidly completing CTA of both lower limbs and confirming the diagnosis of Gustilo type IIIC injury of the right thigh, the patient was given an MESS score of 11. To preserve the patient’s life and avoid acute renal failure and subsequent multiorgan failure after replantation, a medical order for amputation of the right lower limb was issued. Before preparing to enter the operating room for surgery, the patient was given a supplemental physical examination, and a pinprick examination of the toes of the right foot revealed the presence of slow oozing of blood at the toe end, suggesting that a certain amount of collateral blood supply still existed in the right lower limb. After our discussion, we concluded that although the posttraumatic timeframe had been nearly 17 hours, there was still some blood supply to the amputated limb due to the presence of better collateral circulation (Figs. 1 and 2), and limb preservation was possible. After fully communicating with the patient and his family, the surgical plan was immediately changed, and limb preservation treatment was proposed.

### 2.3. Surgical procedure

**Figure 3.**
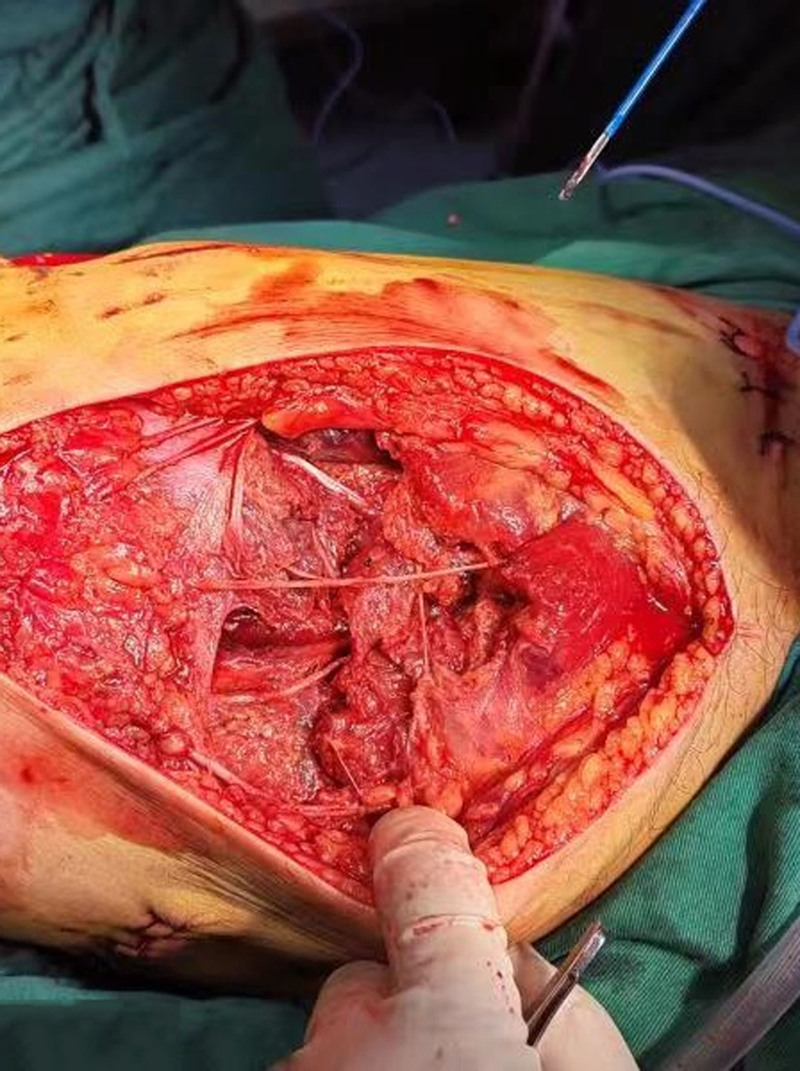
Intraoperative exploration: complete rupture of the thigh muscle.

**Figure 4.**
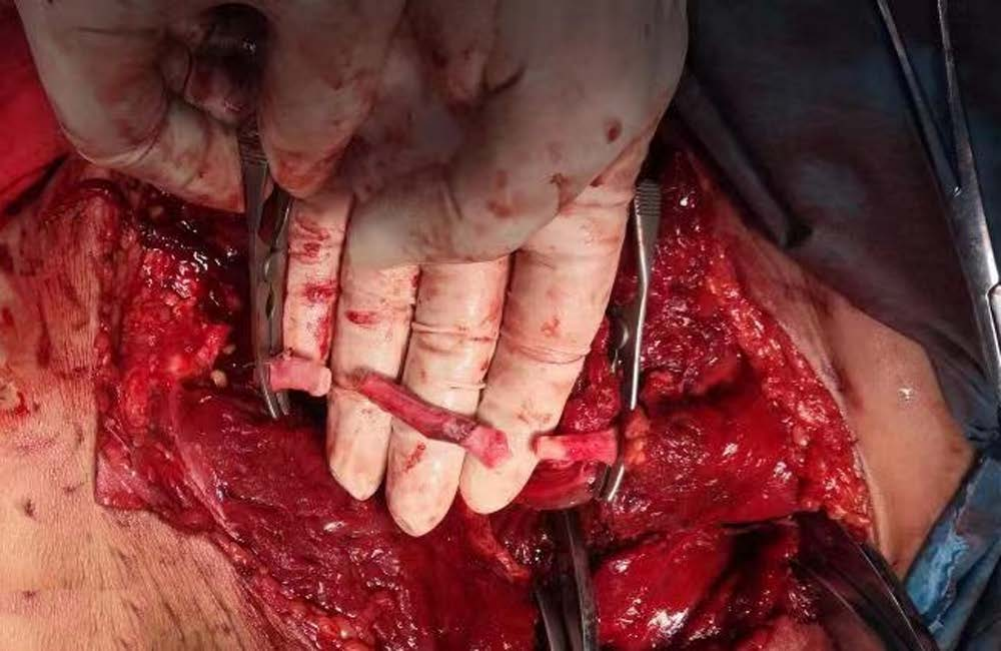
Resection of the injured femoral artery, with sufficient arterial release, allowing for direct anastomosis of a 6 cm defect.

**Figure 5.**
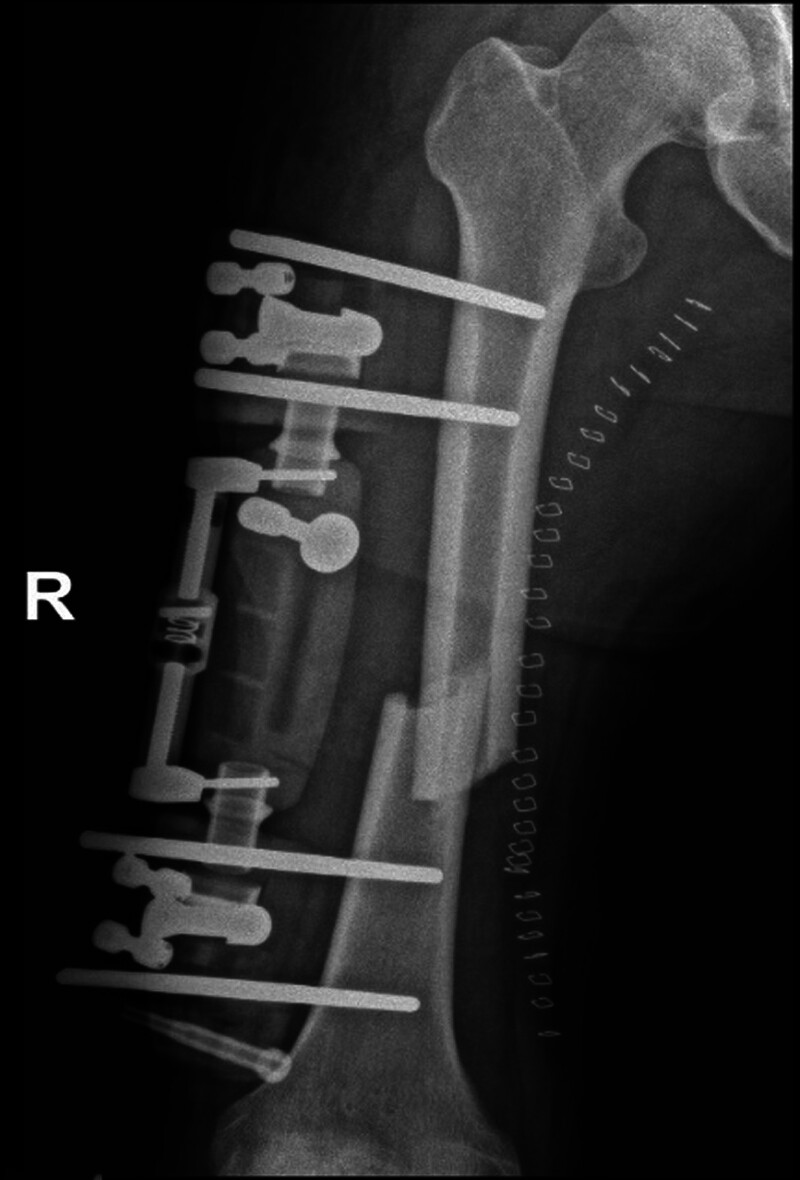
One-stage limb shortening, with the limb fixed in an external fixation frame.

A simple fracture of the middle and lower part of the right femur was detected, and the thigh muscle was completely disrupted at the level of the fracture surface (Fig. 3). The sciatic nerve was not significantly damaged, and the middle and upper part of the right femoral artery were embolized, with an embolus approximately 11 cm in length. The wall of the femoral artery was contused for approximately 4 cm. Resection of the injured segment of the femoral artery with a defect of 6 cm was performed (Fig. 4). Immediately, both ends of the femoral artery were adequately freed, and the muscles on either side of the fractured femoral break starting from the femoral thick line and the bony surface of the femur were sharply stripped partially, after which the femoral fracture ends were overlapped and shortened immediately by about 5 cm, and the fractured ends was stabilized by the use of an external fixation frame with lengthening function (Fig. 5). The defective femoral artery was then anastomosed directly end to end, and the normal blood supply was immediately restored to the distal thigh (revascularization took place nearly 19 hours after the time of injury). The femoral vein was explored for continuity and patency, and no further treatment was performed. Examination showed slightly high tension in the right calf and prophylactic fascial compartment dissection was not performed. The severed muscle group was repaired with sutures. Postoperatively, the patient returned to the intensive care unit for further treatment.

The postoperative shortened right lower limb was successfully hemodynamically reconstructed, and the postoperative indexes of recovery are shown in Table 1.

**Table 1:** Data on the recovery of indicators of the patient’s body after surgery.

Variant	Myoglobin (ng/mL)	Creatine kinase isoenzyme (ng/mL)	Peripheral oxygen saturation at the toes (%)	Urine volume (mL)	Urine color	Renal function	Dorsalis pedis and posterior tibial artery pulsation	Right calf tone
Postoperative day 1	>4095.00	>291.00	96.1	1710	Brown	Normal	Faint	Higher
Postoperative day 2	>3000	144.89	97.6	2230	Brown (darker than before)	-	Faint	Slightly elevated but acceptable
Postoperative day 3	>4095.00	61,25	99.0	2340	Brown (lighter than before)	Normal	Faint.	Slightly elevated but acceptable
Postoperative day 4	2963. 40	51.12	100	2450	Brown (lighter than before)	-	Faint but clearly accessible	Slightly elevated but acceptable
Postoperative day 5	1601.00	-	100	2500	Brown (lighter than before)	Normal	Weak but clearly accessible	lower slightly
Postoperative day 6	-	-	100	2360	Yellow	-	Weak but clearly accessible	lower slightly
Postoperative day 7	607.00	-	-	2620	Yellow	Normal	Clearly reachable	lower slightly
Postoperative day 8	-	-	-	-	Light Yellow	-	Clearly accessible	lower slightly
Postoperative day 9	290	-	-	-	Light yellow	-	Normal.	Slightly higher than normal

**Figure 6.**
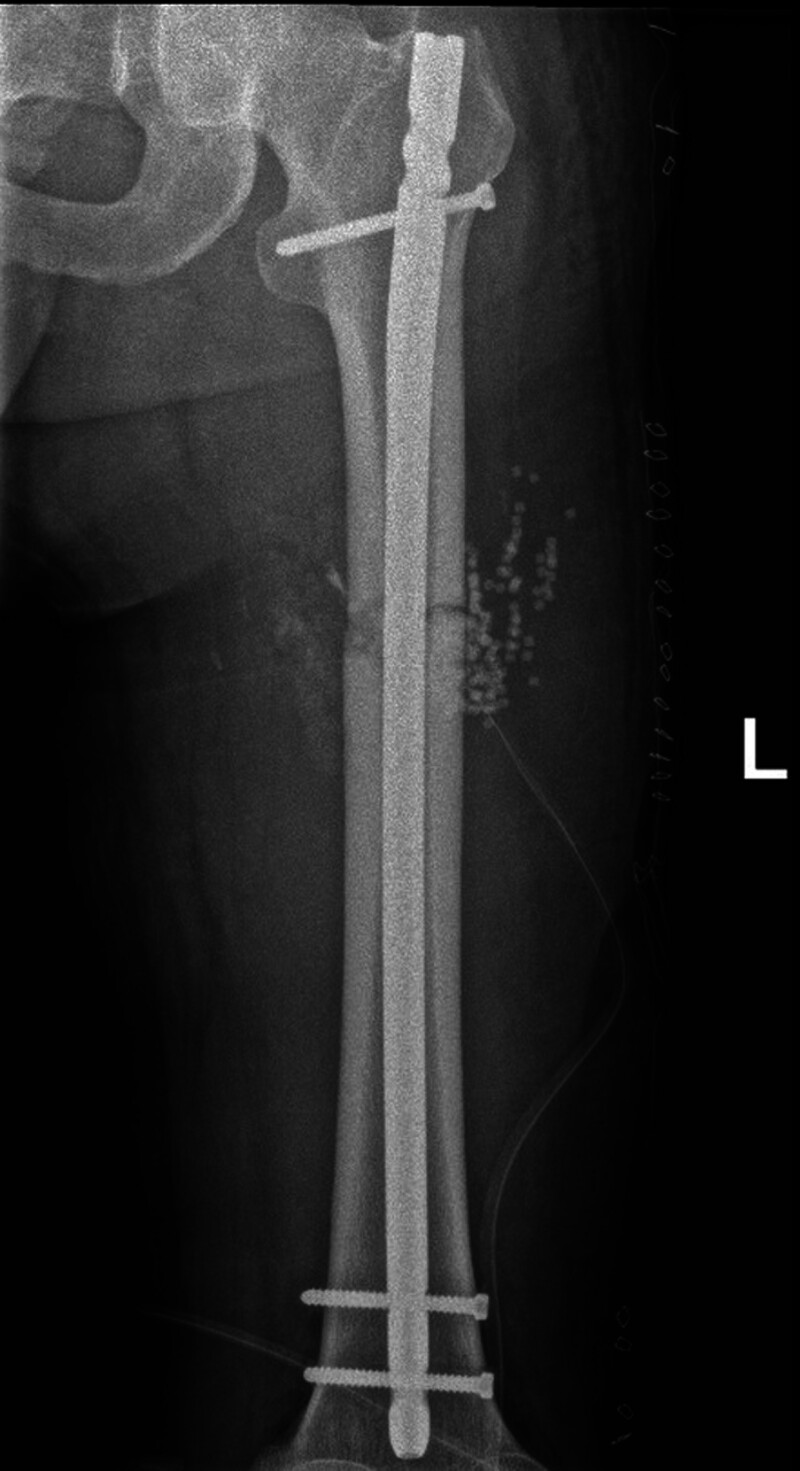
Left femoral internal fixation performed 2 weeks after patient stabilization.

**Figure 7.**
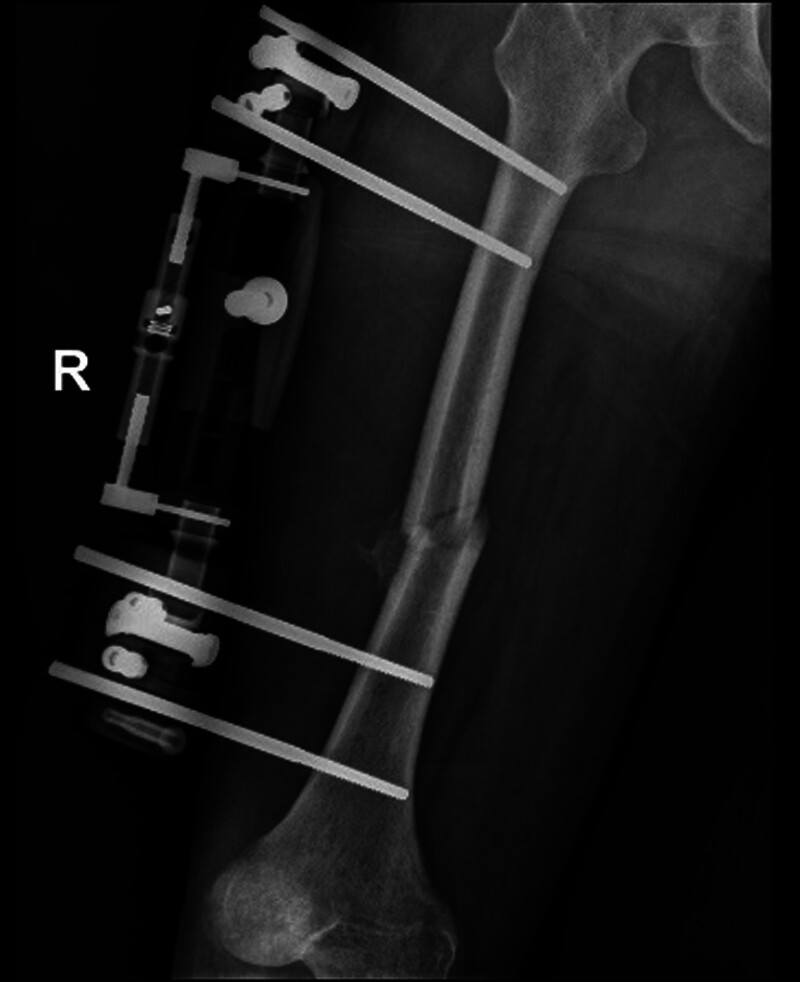
Distraction to restore right femoral length initiated 2 months after successful replantation.

**Figure 8.**
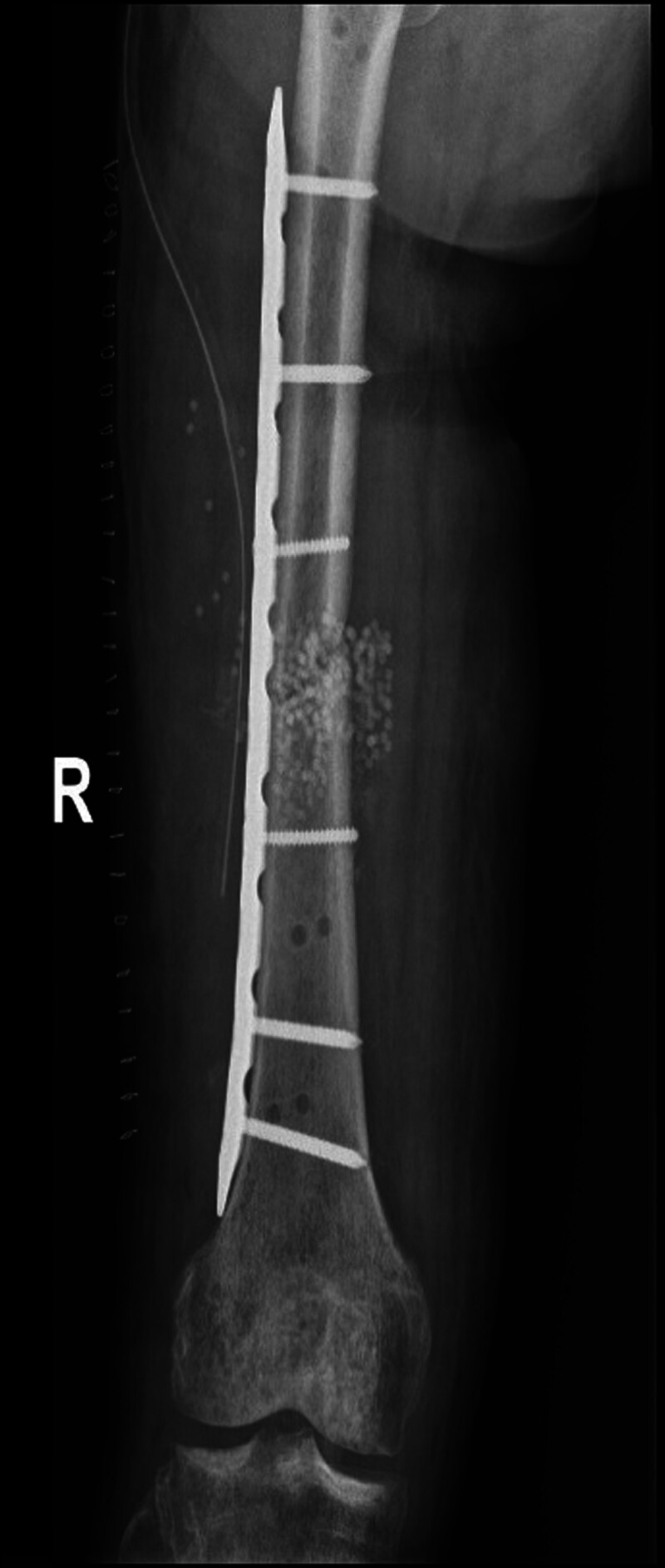
Reduction and internal fixation of the right femur.

Half a month after replantation of a severed right lower limb, intramedullary nail fixation was performed on the left femoral stem fracture (Fig. 6). Two months after right thigh replantation, lengthening of the overlapped and fixed right femur, shortened femoral artery, and piled-up thigh muscles was started by 1 mm per day in 4 times. After 40 days, most of the overlapped femur had disappeared (Fig. 7). The dorsalis pedis and posterior tibial arteries of the right foot were pulsing normally, with good peripheral blood flow. The external fixation frame was removed, and after the needle path was clean, an elective (3.3 months after replantation) internal fixation of the right femoral stem fracture with incision and reduction plate was performed (Fig. 8). Postoperatively, the patient had stiffness in the right hip, right knee, and right ankle joints, and was hospitalized in the rehabilitation department several times, insisting on right lower extremity rehabilitation exercise treatment.

At the last follow-up (16.6 months after replantation), the patient was able to walk freely, with mild claudication (see Supplemental Video 1, Supplemental Digital Content, https://links.lww.com/MD/O676, which demonstrates the patient’s walking status), mild horseshoe foot deformity of the right foot, able to do deep squatting on his own (Fig. 10), squatting down and getting up easily (see Supplemental Video 1, Supplemental Digital Content, https://links.lww.com/MD/O676, which demonstrates the patient squatting), able to climb up and down the stairs without holding the stair handrail easily (see Supplemental Video 1, Supplemental Digital Content, https://links.lww.com/MD/O676, which demonstrates ascending and descending stairs), and able to walk 4 km or run 0.5 km at a time with relative ease. The patient’s right ankle stiffness, and active and passive range of motion were 5°, and the specific right lower limb sensation and muscle strength recovery data are shown in Table 2.

**Table 2:** Sensory and muscle strength recovery data of the patient’s right lower limb.

Variant	Sensation of the right lower limb below the cross-section	Right knee flexor strength	Right knee extensor strength	Right ankle plantarflexor strength	Right ankle dorsiflexor strength
Postoperative day 1	Completely missing	Level 0	Level 0	Level 0	Level 0
Postoperative day 3	Completely absent	Level 0	Level 0	Level 0	Level 0
7th day after surgery	Sensation is returning, numbness is noticeable	Level 0	Level 0	Level 0	Level 0
2 months after surgery	Numbness is noticeably reduced, tactile sensation begins to return	Level 3	Level 3	Level 0	Level 0
5.5 months after surgery	Numbness disappears, tactile sensation is noticeably restored	Level 4	Level 4	Level 1	Level 0
7.5 months after surgery	Sensation is restored more than before	Level 4	Level 4	Level 2	Level 0
9.5 months after surgery	Further recovery of sensation	Level 4	Level 4	Level 3	Level 1
12.5 months after surgery	Further recovery of sensation	Level 5	Level 5	Level 4	Level 2
14.6 months after surgery	Sensation mostly restored, plantar sensation slightly poor, dorsal sensation poor	Level 5	Level 5	Level 4-5	Level 3
16.6 months after surgery	Sensation mostly restored, plantar sensation slightly poor, dorsal sensation poor	Level 5	Level 5	Level 5	Level 3

**Figure 9.**
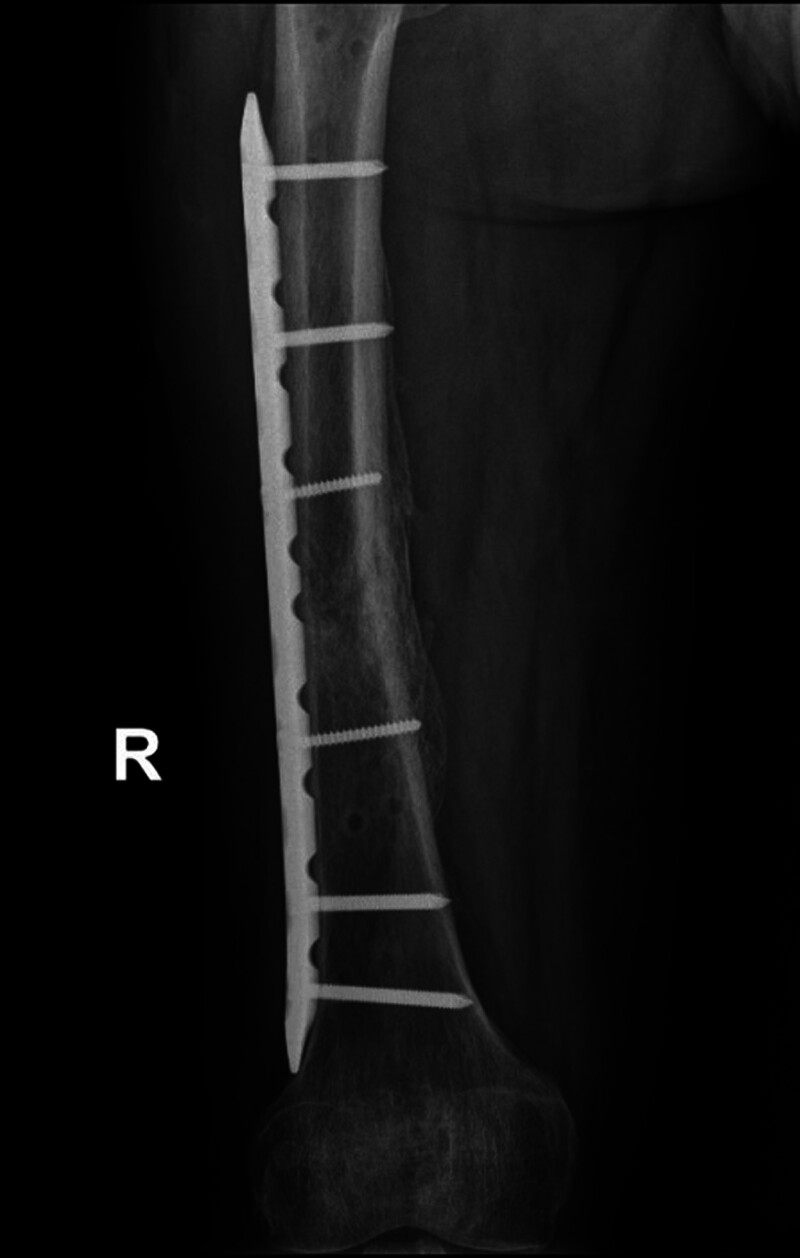
Good healing of the right femur.

**Figure 10.**
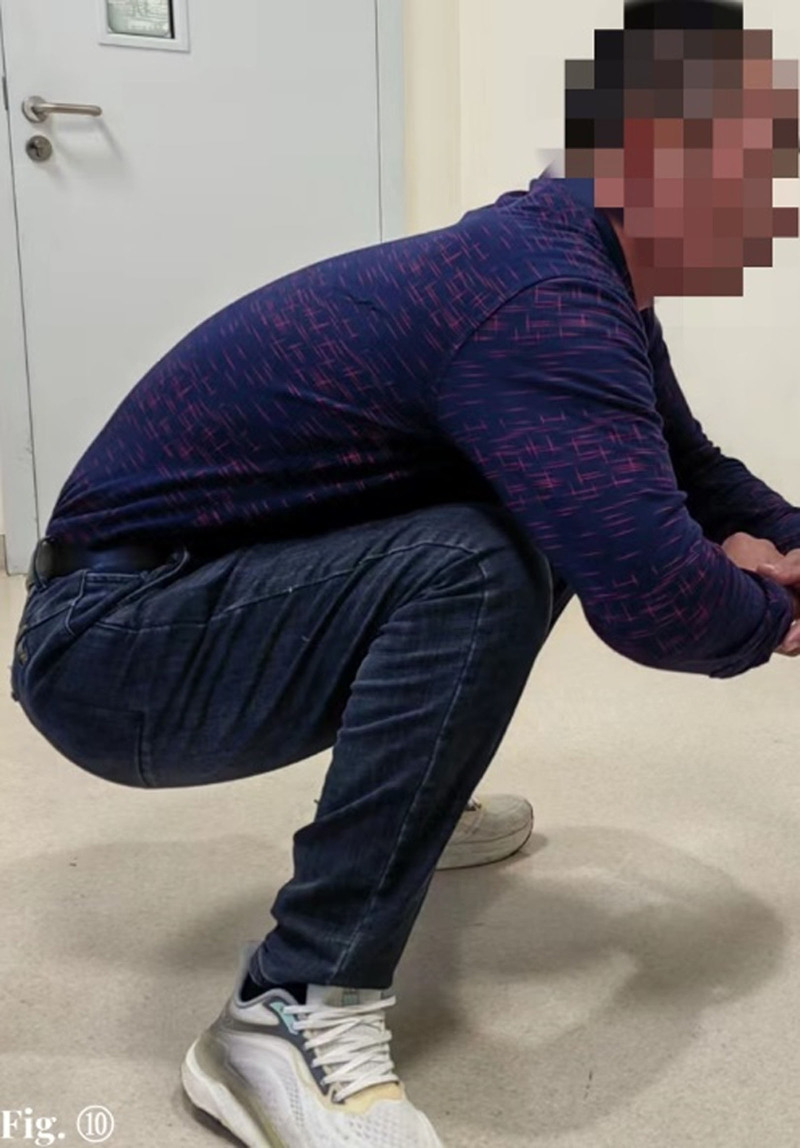
Sixteen and a half months postreplantation, the patient demonstrates improved right lower limb function, with significant sensory recovery and the ability to squat without difficulty.

## 3. Results and evaluation

Bone healing and functional recovery of the lower limbs were evaluated according to the standards of the Association for the Study and Application of Methods of Ilizarov.^[8]^ The right femoral bone healing was evaluated as “Excellent,” the right knee joint function was evaluated as “Excellent,” and the right ankle joint function was evaluated as “Fair.”

The right knee joint was evaluated as “Excellent” according to the Lysholm and Gillquist knee scoring criteria and the Bristol knee scoring system.

The above evaluation results showed that the patient’s recovery was generally good (see Supplemental Videos 1–3, Supplemental Digital Content, https://links.lww.com/MD/O676; https://links.lww.com/MD/O677; https://links.lww.com/MD/O678, which demonstrates the patient’s good recovery of limb function).

## 4. Discussion

### 4.1. Diagnosis of vascular injury

After a limb fracture, clinicians should routinely palpate the terminal artery of the ipsilateral limb and dynamically check for changes in arterial pulsation. In the future, if the arterial pulsation is weakened or disappears and the peripheral blood flow becomes poor, arterial injury should be highly suspected, and CTA or ultrasound should be performed immediately for a clear diagnosis.^[9,10]^

### 4.2. Limb preservation versus amputation in Gustilo type IIIC injuries

Gustilo type IIIC injuries, where making decisions about amputation and limb preservation is complex and difficult.^[11]^ The MESS has been validated by a large body of literature and is the most widely accepted scoring system for guiding therapeutic decision-making in severe limb injuries. Amputation is recommended for trauma patients when their MESS score is greater than or equal to 7 (7–8, relative contraindication to limb-preserving reconstruction; 9–10 and above, absolute contraindication to limb-preserving reconstruction).^[5–7]^

Previous studies have shown that the severity of plantar sensory loss, limb ischemia, and soft tissue injury are the most important factors in determining whether a limb should be preserved.^[11]^ According to Lange, an ischemic time >6 hours is an absolute indication for amputation of a limb in severe trauma.^[12]^ Because the limb is completely ischemic for more than 6 hours, anaerobic respiration for too long, muscle necrosis has occurred, once forced to preserve the limb, after blood circulation, a large number of acidic and toxic substances back to the systemic organs will cause a devastating blow, and ultimately the occurrence of renal failure, multiorgan failure, endangering the patient’s life safety. For this reason, for the main blood vessels to stop blood supply for more than 6 hours of limb preservation, should be strictly controlled, to avoid forced limb preservation and endanger the patient’s life safety. However, this absolute indication for amputation has been questioned, but the longer the ischemic time, the higher the risk of amputation is unquestionable.^[13,14]^ Combined with the success of this case, we believe that the duration of ischemia is a very important indicator, but it is more important to carefully screen out whether the limb still has a partially effective blood supply, and try to avoid amputation of the limb that can be saved.

In our patient, the ischemic time of the lower extremity had been nearly 16 hours at the time of admission, dorsalis pedis, posterior tibial artery could not be touched, the skin of the foot was wet and cold, pale in color, sensation was completely absent below the mid-right thigh, and there was a complete loss of foot and ankle motion, and the MESS score had reached 11, which was strongly in line with the indications for amputation. However, our supplemental examination, with a syringe needle prick of the toe of the right foot, and the slow oozing of blood from the toe, was the only thing that prompted us to change the amputation to limb preservation! We determined that although the patient had interrupted blood flow in the right femoral artery, the patient’s robust collateral circulation provided a valuable volume of blood flow to the distal limb (Figs. 1 and 2).^[5,7,10,15,16]^ The patient’s ultimate success in preserving the limb and obtaining a relatively desirable limb function (see Table 2) proved that our judgment was correct. In our search of the previous literature, we did not find studies or case reports of slow oozing of blood after needling the end of a limb as an indication for limb preservation, nor did we find reports of ischemia longer than 19 hours with successful limb preservation, and our report provides experience and confidence for other colleagues in making decisions about limb preservation in the face of prolonged ischemic limbs.

Our case shows that even if the ischemia lasts for a very long time, even if there is a complete loss of sensation and movement at the distal end of the limb, as long as there is still a part of effective blood supply to the distal end of the limb, the restoration of sensation and movement function of the limb is possible.

### 4.3. Rapid restoration of blood supply: 1-stage limb shortening, 2-stage lengthening

In order to revascularization as quickly as possible, we applied the Ilizarov technique, which was previously used mainly for repairing large bone defects or soft tissue defects, to vascular repair in patients with vascular defects but no bone defects.^[17]^ First, the muscles on either side of the fractured femoral break starting from the femoral thick line and the bony surface of the femur are sharply stripped to allow the femur to shorten sufficiently to allow direct anastomosis of the femoral artery in the presence of a long segmental defect. In our patient, the femur was immediately shortened by approximately 5 cm and stabilized with an external fixation frame (Fig. 5). Due to adequate freeing of both sides of the femoral artery, the femoral artery, which had a defect of up to 6 cm, was easily anastomosed end to end, and the 1-time access to the revascularization was successful. This ensured that normal blood supply was restored to the distal limb in the shortest time, shortened the ischemic time, avoided necrosis of the distal limb, and created conditions for the survival of the limb, the maximum possible restoration of sensory and motor functions, and the avoidance of calf prophylactic fascial compartment incision and decompression. Waiting for the success of limb preservation, then phase II gradually pull apart the overlapping femur, shortened thigh muscle groups, nerves, and other soft tissues (Fig. 7), the focus was on pulling the femoral artery, which had a defect up to 6 cm, to normal length. The stretching process was safe as only 1 mm was stretched per day. After the femoral artery had regained its normal length, the femoral fracture was reset and fixed with a plate. Eventually, the limb preservation was successful, the fracture healed well, and a normal length limb and bony architecture were obtained (Fig. 9).

### 4.4. Does the calf need to be decompressed by prophylactic fasciotomy?

In patients with large vessel injury to the limb and interruption of blood flow, ischemia-reperfusion injury is very likely to occur after reestablishment of blood flow and osteofascial compartment syndrome is a common type of limb ischemia-reperfusion injury. Once osteofascial compartment syndrome occurs, if it is not detected in time, it can lead to necrosis of the patient’s limbs and renal failure, which can jeopardize the patient’s life. In view of the serious consequences of osteofascial compartment syndrome, previous opinions have advocated prophylactic fasciotomy.^[18,19]^ With further research, more and more scholars believe that only a minority of patients with extremity vascular trauma require fasciotomy.^[20]^ There are reports that fasciotomy also increases long-term venous complications, as well as affects the muscle pump function of the gastrocnemius muscle.^[21,22]^ This evidence suggests that aggressive prophylactic fasciotomy may not be necessary in some patients with osteofascial compartment syndrome in the clinical setting, but further clarification is needed regarding the indications for conservative treatment of osteofascial compartment syndrome, and standardized conservative treatments need to be proposed.^[23,24]^ In our patient, although the thermal ischemia time was nearly 19 hours, there was still a slow oozing of blood from the toe pinprick. Considering that the sound collateral circulation ensured that part of the blood supply still existed far from the severed end, and the anaerobic respiration of the distal limb was not complete, and the tension in the distal calf was not very high, after revascularization, the tension in the distal calf did not rise rapidly, so we chose not to cut open the osteofascial compartment. Closely observe the change of calf tension, once the tension increased significantly, then quickly perform the calf osteofascial compartment incision and decompression. In the end, our judgment was accurate, and the patient survived the ischemia-reperfusion injury.

### 4.5. Prevention of joint stiffness – early rehabilitation exercise

Even if replantation of a severed limb successful, ischemic myoclonus occurs to varying degrees due to prolonged muscle ischemia, and coupled with intra-articular adhesions, most replanted limbs will experience severe joint stiffness. In order to prevent the occurrence of joint stiffness, early passive joint functional exercises as well as late active functional exercises are particularly important.^[25,26]^ In our patient, due to the adherence to early passive plus late active functional exercises, he eventually gained essentially normal hip and knee function, and his ankle joint stiffness was better recovered (Fig. 10 and Supplemental Videos 1–3, Supplemental Digital Content, https://links.lww.com/MD/O676; https://links.lww.com/MD/O677; https://links.lww.com/MD/O678).

## 5. Conclusion

In summary, for Gustilo IIIC injury with ischemic time limit more than 6 hours, the decision of amputation or limb preservation should be made carefully, and it is crucial to determine whether there is a small amount of effective perfusion in the distal limb, and whether oozing of blood occurs by pinprick at the end of the limb can be used as an important reference index; the use of Ilizarov pulling and regeneration technology can shorten the limb immediately to quickly restore blood supply to the distal limb, and then pulling to restore the length of vessels with defects can increase the success rate of limb preservation. The success rate of limb preservation can be improved by restoring the length of the defective blood vessels with Ilizarov pulling; the prophylactic osteofascial compartment dissection of the distal limb after reimplantation should be flexibly grasped; active limb rehabilitation exercises after successful limb preservation play an important role in improving the function of the limb.

## Acknowledgments

We would like to thank the patient for generously permitting the use of the data in this report.

## Author contributions

**Software:** Qiushun Zhang.

**Writing – original draft:** Qiushun Zhang, Guangying Zhang.

**Methodology:** Feng Zhang.

**Writing – review & editing:** Feng Zhang, Dongmei Li, Yifeng Zhao.

**Resources:** Wenzhuo Li, Yifeng Zhao.

**Supervision:** Wenzhuo Li, Dongmei Li, Yifeng Zhao.

**Conceptualization:** Yifeng Zhao.

## Supplementary Material


